# Rev-erbα heterozygosity produces a dose-dependent phenotypic advantage in mice

**DOI:** 10.1371/journal.pone.0227720

**Published:** 2020-05-14

**Authors:** Ryan D. Welch, Cyrielle Billon, Amina Kameric, Thomas P. Burris, Colin A. Flaveny

**Affiliations:** 1 The Salk Institute for Biological Sciences, La Jolla, CA, United States of America; 2 The Center for Clinical Pharmacology, Saint Louis College of Pharmacy, Saint Louis, MO, United States of America; 3 The Department of Pharmacology and Physiology, Saint Louis University School of Medicine, Saint Louis, MO, United States of America; 4 The Alvin J. Siteman Cancer Center at Barnes-Jewish and Washington University School of Medicine in Saint Louis, Saint Louis, MO, United States of America; 5 The Saint Louis University Henry and Amelia Nasrallah Center for Neuroscience, Saint Louis, MO, United States of America; Tohoku University, JAPAN

## Abstract

Numerous mutational studies have demonstrated that circadian clock proteins regulate behavior and metabolism. *Nr1d1(Rev-erbα)* is a key regulator of circadian gene expression and a pleiotropic regulator of skeletal muscle homeostasis and lipid metabolism. Loss of *Rev-erbα* expression induces muscular atrophy, high adiposity, and metabolic syndrome in mice. Here we show that, unlike knockout mice, *Nr1d1* heterozygous mice are not susceptible to muscular atrophy and in fact paradoxically possess larger myofiber diameters and improved neuromuscular function, compared to wildtype mice. Heterozygous mice lacked dyslipidemia, a characteristic of *Nr1d1* knockout mice and displayed increased whole-body fatty-acid oxidation during periods of inactivity (light cycle). Heterozygous mice also exhibited higher rates of glucose uptake when fasted, and had elevated basal rates of gluconeogenesis compared to wildtype and knockout littermates. *Rev-erbα* ablation suppressed glycolysis and fatty acid-oxidation in white-adipose tissue (WAT), whereas partial *Rev-erbα* loss, curiously stimulated these processes. Our investigations revealed that *Rev-erbα* dose-dependently regulates glucose metabolism and fatty acid oxidation in WAT and muscle.

## Introduction

Circadian rhythmicity directs many aspects of behavior and metabolism [1]. The circadian clock is controlled by interconnected proteins that oscillate in activity and expression over a 24-hour cycle [2]. The central/master regulator of the internal clock is located within the suprachiasmatic nucleus (SCN) of the hypothalamus and integrates environmental light cues to synchronize peripheral oscillators in the liver, pancreas, and skeletal muscle (SkM) [1]. In addition to the central circadian clock, peripheral clocks can undergo autonomous oscillatory shifts in response to changes in nutrient availability without input from the central clock [3–6]. In all tissues the core molecular clock activators BMAL1 and CLOCK induce the transcription of circadian regulators PER and CRY that in turn inhibit CLOCK and BMAL1 activity, creating consistently robust diurnal oscillations [7]. This molecular clock has an auxiliary loop that is modulated by the nuclear receptors Retinoid-receptor like orphan receptor (ROR) and REV-ERBα/β (NR1D1/2) [8]. ROR, a transcriptional activator and REV-ERB a constitutive transcriptional repressor, reciprocally modulate each other’s activity by binding competitively to the same REV-ERB response elements (RevREs). Each receptor therefore oppositely regulate expression of the clock proteins BMAL1 and CLOCK, and therefore form an additional regulatory arm to the core molecular clock that enables more robust control of behavior in response to metabolic cues [8–10].

Disruptions of circadian rhythm is a well-characterized contributary factor to metabolic disorders in humans [1]. Mutational studies in mice have demonstrated that *Clock* mutation promotes obesity and metabolic syndrome characterized by hyperlipidemia, hyperleptinemia, hepatic steatosis, hyperglycemia, and deficient insulin production [11]. *Bmal1* mutant studies have demonstrated the importance of the molecular clocks in mitochondrial dynamics and type-2 diabetes protection [5, 12]. Additionally, the loss of *Bmal1* in mice is known to produce symptoms of advanced aging, including total body weight loss and sarcopenia [13]. REV-ERBα, like other circadian factors, is a key metabolic regulator of energy homeostasis. *Rev-erbα* deficient mice have higher basal glucose levels, enhanced fatty-acid synthesis-gene expression and display shifts in metabolic substrate preference in response to specific changes in light/dark cycles [14]. Moreover, *Rev-erbα* deficient mice display a reduction in lipid mobilization under fasted conditions and are consequently susceptible to high adiposity [14, 15].

REV-ERB has a pivotal role in muscle homeostasis as well. Previously, we have highlighted that REV-ERBα directs myogenesis through tethered interaction with the transcription complex Nuclear Factor-Y [16]. Moreover, inhibiting REV-ERB with a synthetic antagonist enhanced muscle repair in an acute injury model and slowed disease progression in a model of Duchenne’s muscular dystrophy [16, 17]. Loss of *Rev-Erbβ* in contrast to *Rev-Erbα* was also shown to stimulate skeletal muscle metabolism and fatty acid oxidation [18]. We have previously noted that *Nr1d1*^*+/-*^ mice had larger myofiber diameters compared to wild-type and *Nr1d1*^*-/-*^ mice, which implied that partial loss of REV-ERBα uniquely stimulated muscle hypertrophy and or inhibited muscle degradation. Heterozygous mice also showed an enhanced regenerative response to injury compared to wildtype mice [16]. *Rev-erbα* null mice are known to display elevated expression of mediators of muscle atrophy, impaired muscle function, and a progressive decline in myofiber size due to enhanced protein catabolism [19]. In order to decipher the metabolic underpinnings of this gene dose-effect we comprehensively characterized the metabolic phenotype of *Nr1d1*^*+/-*^ mice. We demonstrate that young *Nr1d1* heterozygous mice have larger myofibers and a greater lean mass than wildtype and knockout littermates. We also show that *Rev-erbα* heterozygosity enhanced lean mass composition and induced fatty oxidation in WAT adipose tissue as well as increased glucose sensitivity and enhances gluconeogenesis compared to *Rev-erbα* null and wildtype mice. Our results illustrate that there is a unique dose-threshold for REV-ERB regulation of metabolic activity. This discovery adds a new layer to the complexity of Rev-erb modulation of muscle homeostasis and lipid metabolism.

## Methods

### Mice

C57BL/6 and B6.Cg-Nr1d1tm1Ven/LazJ (*Nr1d1*^+/-^) mice were purchased from the Jackson laboratory and these mice were used founders for an inbred colony of mice that was used to generate *Nr1d1*^+/+^, *Nr1d1*^-/-^ and *Nr1d1*^+/-^ genotypes. All experiments shown includes cohorts of age-matched littermates. All mice were housed in a 12h-12h light-dark cycle, received a standard chow diet, and were allowed food and water *ad libitum*. At the conclusion to the study mice were sacrificed by CO_2_ asphyxiation followed by cervical dislocation. Mouse experimental procedures were approved by the Saint Louis University Institutional Animal Care and Use Committee (protocol#2474). Mouse sample size (n) was chosen using an α set at 0.05 a priori with a power of 80.

### NMR Body composition analysis

Nuclear magnetic resonance (NMR) analyses were conducted once a week on age matched 6-week old male *Nr1d1*^+/+^, *Nr1d1*^+/-^, and *Nr1d1*^-/-^ mice (n = 7 per group) until 12 weeks of age by utilizing a Bruker BioSpin LF50 Body Composition Analyzer. Statistically significant differences in lean, and fat mass were determined by 2-way ANOVA with an alpha set at 0.05.

### H&E staining and cross-sectional area (CSA) analysis

Muscle samples (Tibialis anterior or gastrocnemius) were fixed overnight in 4% formalin and then embedded into paraffin. Paraffin embedded muscle sections where stained by a standard hematoxylin and eosin (H&E) protocol. Slide images were taken by a Leica MC120 HD attached to a Leica DM750 microscope. Cross-sectional area (CSA) of H&E stained myofibers where quantified by ImageJ. Six images from each experimental animal was utilized to calculate the average CSA per mouse. Images were taken at 10x and 40x for needed calculations.

### Grip strength

Hindlimb and forelimb grip strength were tested simultaneously with a digital force instrument (BIOSEB) as described previously [20]. Each (*Nr1d1*^*+/+*^, *Nr1d1*^*+/-*^ or *Nr1d1*^*-/-*^) mouse was subjected to 3 grip strength tests at 5 weeks and 11 weeks of age with a minimum of 10-minute rest intervals between each test. To reduce user-specific bias, two distinct researchers administered tests. Values from the two separate experimental groups were recorded and pooled. Statistical significance between the respective groups was determined using a student’s t-test with an alpha set at 0.05.

### Quantitative RT-qPCR

Total RNA was isolated from muscle, eWAT and liver tissue using Trizol reagent (Invitrogen). Isolated RNA (1μg) was reverse-transcribed into cDNA using qScript cDNA Synthesis Kit (Quanta Biosciences, Inc.). Quantitative PCR (qPCR) was performed with SYBR Select Master Mix (Applied Biosystems) and cognate primers. Gene expression levels for mouse tissue and cells were normalized to *Gapdh*.

### Immunoblotting

Cells and muscle tissues were lysed by a RIPA with protease inhibitor buffer. The extracted proteins were flash frozen and stored at -80°C. The extracted proteins were separated by SDS-PAGE and transferred onto PVDF membranes. Immunoblot analyses were performed with standard procedures.

### Feeding study

Feeding behavior in 8-week-old *Nr1d1*^*+/+*^ and *Nr1d1*^*+/-*^ mice was assessed over 14 days using the BioDAQ episodic intake monitor. Mice were allowed food and water *ad libitum*. Data was collected and analyzed using BioDaq software. Statistically significant differences in daily food intake, daily water intake, and daily feeding events for each group of mice were determined using a student’s t-test with an alpha set as 0.05.

### Wheel-running activity

*Nr1d1*^*+/+*^ and *Nr1d1*^*+/-*^ (n = 6 per group) mice wheel-running activity were monitored for 4 weeks using wheel-running cages (Actimetrics) in circadian cabinets. For light/dark experiments, mice were kept on a strict 12h-12h light dark cycle mice. For dark/dark experiments, mice were kept in 24h darkness. The activity data was collected and actograms were generated using ClockLab (MatLab) software.

### Glucose tolerance test (GTT)

Mice were fasted for 6 hours prior to GTT. Each mouse was weighed and the needed dose of glucose calculated. Glucose was prepared in a glucose/PBS solution that contained 250mg/ml of glucose. Injection volume was calculated by BW(g) X 10uL of PBS. Fasting blood glucose was measured using a Glucometer (One Touch Ultra^™^) by tail bleeds. Blood glucose was measured at 15, 30, 60, and 120 minutes’ post glucose injections.

### Insulin tolerance test (ITT)

Mice were fasted for 6 hours prior to ITT. Each mouse was weighed and the needed dose of insulin calculated. Insulin was prepared in an insulin/PBS solution that contained 0.1U/ml of insulin. Injection volume was calculated by BW(g) X 10uL of PBS. Fasting blood glucose was measured using a Glucometer (One Touch Ultra^™^) by tail bleeds. Blood glucose was measured at 15, 30, 60, and 120 minutes’ post insulin injections.

### Pyruvate tolerance test (PTT)

Mice were fasted for 6 hours prior to PTT. Each mouse was weighed and the needed dose of pyruvate calculated. Pyruvate was prepared in a pyruvate/PBS solution that contained 100mg/ml of pyruvate. Injection volume was calculated by BW(g) X 10uL of PBS. Fasting blood glucose was measured using a Glucometer (One Touch Ultra^™^) by tail bleeds as above. Blood glucose was measured at 15, 30, 60, and 120 minutes’ post injections.

### Metabolic cages

Whole body metabolic states were tested by indirect calorimetry in a Comprehensive Lab Animal Monitoring System (Columbus Instruments) for 3 days after 5 days of habituation at 22°C (room temperature) or 30°C (thermoneutrality). CLAMS operation and analysis were conducted as recommended by the manufacturer. Age matched mice (12–16 weeks old) were single housed and light and feeding conditions mirrored home cage conditions. The respiratory exchange ratio (RER) was calculated by VCO2 and VO2 as determined by the light/dark cycle. VCO2, VO2, and heat production values were normalized to lean mass.

### Statistical analysis

Statistical significance was determined by subjecting mean values per group to students-t test, One-Way ANOVA, Two-Way ANOVA unless otherwise specified. A value of p≤0.05 is considered statistically significant.

## Results

### Rev-erbα expression modulates body composition and muscle function

**Fig 1:**
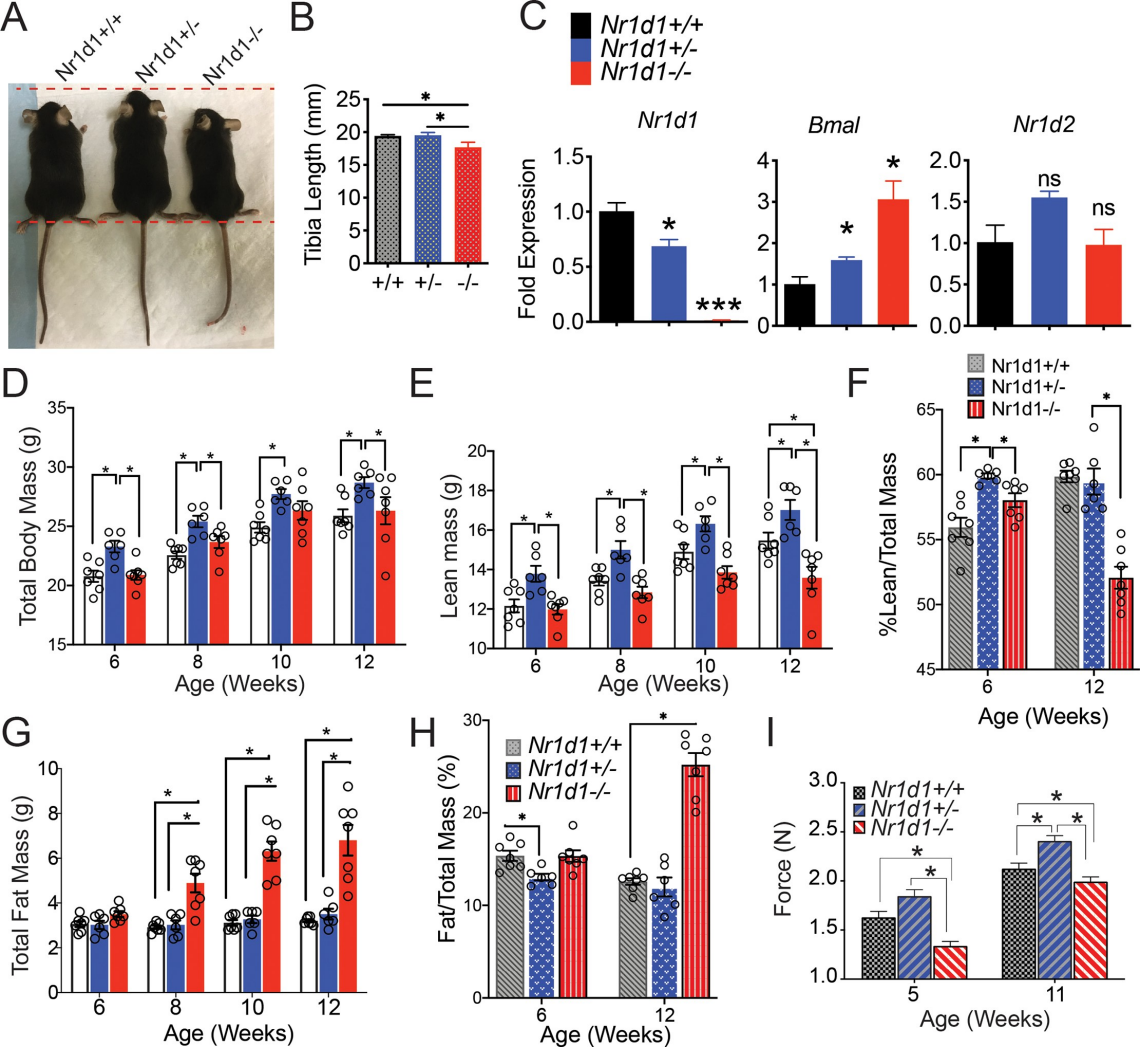
*Nr1d1* expression paradoxically modulates body composition and grip strength in mice. (a) Representative sizes of *Nr1d1*^*+/+*^, *Nr1d1*^*+/-*^ and *Nr1d1*^*-/-*^ mice. Mice pictured are 6-week-old male littermates. (b) Mean length of left tibia bones isolated from *Nr1d1*^*+/+*^, *Nr1d1*^*+/-*^ and *Nr1d1*^*-/-*^ mice. (c) *Rev-erbα* and *Bmal1* mRNA transcripts in skeletal muscle of *Nr1d1*^*+/+*^, *Nr1d1*^*+/-*^ and *Nr1d1*^*-/-*^ at 5–7 weeks of age as determined by RT-qPCR (n = 4) (d) Total body weight of *Nr1d1*^*+/+*^, *Nr1d1*^*+/-*^ and *Nr1d1*^*-/-*^ at 6–12 weeks of age. (e) Total lean mass and (f) percent lean mass of *Nr1d1*^*+/+*^, *Nr1d1*^*+/-*^ and *Nr1d1*^*-/-*^ at 6–12 weeks of age (g) Fat mass and (h) percent fat mass of 6-12-week old *Nr1d1*^*+/+*^, *Nr1d1*^*+/-*^ and *Nr1d1*^*-/-*^mice. Lean and fat mass +/- standard deviation were determined by using a Bruker BioSpin LF50 Body Composition Analyzer (n = 7). Statistically significant differences in lean, fat and fluid mass were determined by a 2-Way ANOVA *p< 0.05. (i). Forelimb and hindlimb grip strength of *Nr1d1*^*+/+*^, *Nr1d1*^*+/-*^ and *Nr1d1*^*-/-*^ mice. Grip strength was measured using a digital force instrument (BIOSEB) (n = 7). Mean grip strength force in N was analyzed using One-Way ANOVA *p<0.05.

In accord with previous studies, we observed that heterozygous REV-ERBα mice in addition to possessing heightened regenerative capacity interestingly had a greater total average body mass than wildtype mice [16] (Fig 1A). The tibia lengths of *Nr1d1*^*+/-*^ compared to wildtype and knockout mice was also significantly larger (Fig 1B). We quantified the expression of *Rev-erbα* in the SkM of *Nr1d1*^*+/+*^, *Nr1d1*^*+/-*^ and *Nr1d1*^*-/-*^ mice at CT8 and found that *Rev-erbα* gene expression was dose dependently linked to *Nr1d1* genotype as partial loss of *Rev-erbα* resulted in a reduction in REV-ERBα expression (Fig 1B). Importantly *Nr1d1*-heterozygous mice did not display a compensatory increase in *Nr1d2* expression. To further ascertain the role of REV-ERBα we assayed the body composition of *Rev-erbα* null (*Nr1d1*^*-/-*^), wild type (*Nr1d1*^*+/+*^) and *Rev-erbα* heterozygous (*Nr1d1*^*+/-*^) mice. Surprisingly, *Nr1d1*^*+/-*^ mice displayed a higher mean body mass compared to that of the wild type and knockout mice from 6 to 12 weeks of age (Fig 1C). Nuclear magnetic resonance (NMR)-based body composition analysis revealed that the enhanced size of heterozygous mice in addition to enhanced skeletal size was also due to a stable elevation of total fat free lean mass (Fig 1D–1F). The percentage lean mass was greater in heterozygous mice up until 12 weeks of age where it then was comparable to that of wildtype mice (Fig 1E and 1F). Interestingly, over time knockout mice exhibited a stark decline in lean mass (Fig 1D–1F). Knockout mice also showed increased adiposity with age, as shown previously [21], (Fig 1G and 1H). Conversely, *Nr1d1*^*+/-*^ mice adiposity was significantly lower than knockout and wildtype mice up until 12 weeks of age when it became indistinguishable from that of wildtype mice (Fig 1G and 1H). Interestingly, *Nr1d1*^*+/-*^ mice of 5 and 11-weeks of age also exhibited superior grip strength when compared to *Nr1d1*^*+/+*^ and *Nr1d1*^*-/-*^ mice (Fig 1I).

These results hinted that Nr1d1 heterozygosity selectively increased lean mass levels and suppressed adipose tissue mass in young mice and suggested that partial *Nr1d1* loss may enhance neuromuscular function.

### Nr1d1 heterozygous mice have normal circadian rhythms

It is known that full loss of *Rev-erbα* expression does not promote changes in wheel running activity in mice that are exposed to normal light/dark cycles [22, 23]. However, *Nr1d1*^*-/-*^ mice subjected to constant darkness display a shift in circadian activity, demonstrating that REV-ERBα is an important regulator of circadian behavior [22]. *Nr1d1*^*+/-*^ mice had normal circadian behavior when compared to wild-type mice in either normal light/dark cycles or in constant darkness, suggesting that the regulation of circadian function is preserved in the *Nr1d1*^*+/-*^ mice (S1A Fig, S1B Fig). Interestingly, this is despite heterozygous mice exhibiting reduced expression of *Rev-erbα* and the significant increase in *Bmal1* expression (Fig 1B). These results collectively suggest that partial loss and homozygous deletion of Rev-erbα has contrasting effects on whole body composition that is not linked to changes in circadian regulation or feeding behavior.

### Heterozygous show enhanced myofiber size that is preserved after maturation

**Fig 2:**
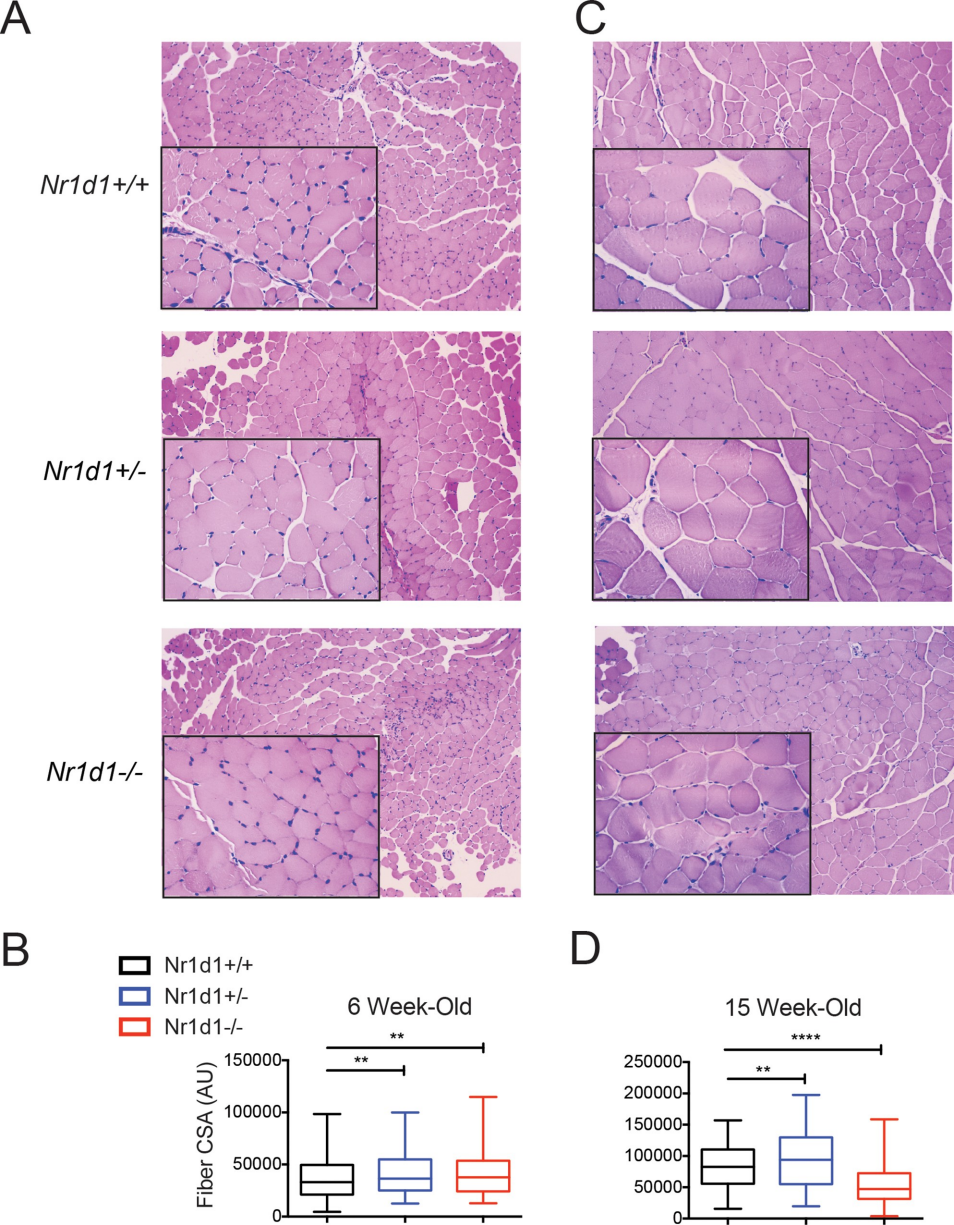
Loss of *Nr1d1* expression increases muscle fiber cross sectional area in young mice but reduces it older mice. (a) Hematoxylin/eosin (H&E) stained tibialis anterior muscle sections from 6 week old *Nr1d1*^*+/+*^, *Nr1d1*^*+/-*^ and *Nr1d1*^*-/-*^ mice (b) Quantification of myofiber cross-sectional area (CSA) from 6 week-old *Nr1d1*^*+/+*^, *Nr1d1*^*+/-*^ and *Nr1d1*^*-/-*^ mice (n = 4) (c) H&E stained tibialis anterior muscle sections from 15 week-old *Nr1d1*^*+/+*^, *Nr1d1*^*+/-*^ and *Nr1d1*^*-/-*^ mice. (d) Quantified CSA from of TA myofibers from 15 week-old *Nr1d1*^*+/+*^, *Nr1d1*^*+/-*^ and *Nr1d1*^*-/-*^ mice. CSA determined using ImageJ software. Units displayed are arbitrary units (AU) error bars +/- standard deviation **p<0.01 and ****p<0.0001 determined by One-Way ANOVA.

As previously mentioned, compared to wildtype littermates null mice show a rapid decline in lean mass after reaching maturity [16]. We analyzed the CSA of tibialis anterior muscle from *Nr1d1*^*+/-*^ and *Nr1d1*^*-/-*^ mice and found that, surprisingly, both knockout and heterozygous mice displayed significantly larger myofibers than wildtype littermates at 6-weeks-of age (Fig 2A and 2B). Interestingly, 15-week old *Nr1d1*^*-/-*^ mice showed progressive muscular atrophy while *Nr1d1*^*+/-*^ mice retained a larger myofiber size than that of wildtype mice (Fig 2C and 2D). To determine the underlying factors driving the distinct phenotypes we measured food/water intake and average number of feeding events in *Nr1d1*^*+/-*^ and *Nr1d1*^*-/-*^ mice. We observed no differences in the amount of food consumed, the frequency, or timing of feedings between *Nr1d1*^*+/+*^, *Nr1d1*^*+/-*^ and *Nr1d1*^*-/-*^ mice (S2A–S2C Fig), suggesting *Nr1d1* heterozygosity did not influence lean mass through augmentation of feeding behavior. These results complement similar observations in *Nr1d1*^*-/-*^ mice that highlighted that complete loss of *Rev-erbα* did not produce changes in feeding activity and food intake when allowed food *ad libitum* [14, 15].

### Partial loss of *Rev-erbα* expression does not alter catabolic or anabolic gene expression in skeletal muscle

**Fig 3:**
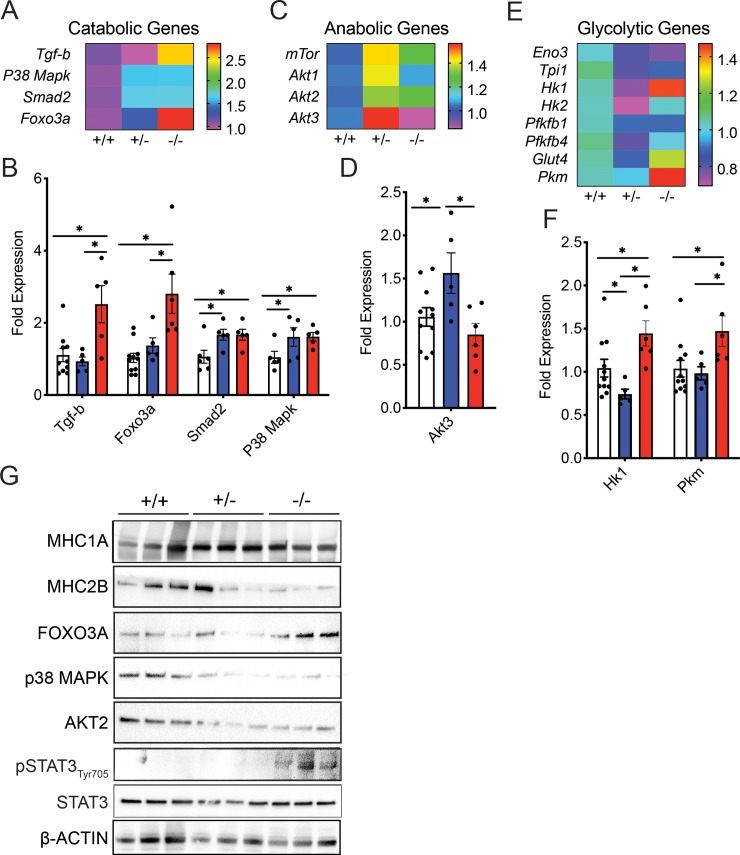
Complete *Nr1d1* ablation induces catabolism of skeletal muscle in older mice. (a) Heat-map showing the RT-qPCR quantified expression pattern of muscle catabolism genes *Tgfb*, *Foxo3a*, *Smad2*, and *p38Mapk* in the soleus muscle of 15-week-old *Nr1d1*^*+/+*^, *Nr1d1*^*+/-*^ and *Nr1d1*^*-/-*^ mice. (b) Mean expression of differentially-regulated catabolic factors in the soleus muscle of 15-week-old *Nr1d1*^*+/+*^, *Nr1d1*^*+/-*^ and *Nr1d1*^*-/-*^ mice shown in (n = 6 error bars +/- stdev) (c) Heat-map showing the RT-qPCR expression pattern of muscle anabolic genes *mTor* and *Akt1-3* in the TA muscle of 15-week-old *Nr1d1*^*+/+*^, *Nr1d1*^*+/-*^ and *Nr1d1*^*-/-*^. (d) RT-qPCR-quantified expression of *Akt3* in the soleus muscle of 15-week-old *Nr1d1*^*+/+*^, *Nr1d1*^*+/-*^ and *Nr1d1*^*-/-*^ mice (n = 6 error bars +/- stdev). (e) Heat-map showing the RT-qPCR quantified expression pattern of muscle glycolytic enzymes *Eno3*, *Tpi1*, *Hk1*, *Hk2*, *Pfkfb1*, *Pfkfb4*, *Glut4* and *Pkm* in the soleus muscle of 15-week-old *Nr1d1*^*+/+*^, *Nr1d1*^*+/-*^ and *Nr1d1*^*-/-*^ mice. (f) Mean expression of differentially-regulated glycolytic enzymes in the soleus muscle of 15-week-old *Nr1d1*^*+/-*^ mice shown in (n = 6 error bars +/- stdev) (g) Immunoblot showing expression of MHC1A (slow myosin heavy chain), MHC2B (fast myosin heavy chain), FOXO3A, P38MAPK, AKT2 and ß-Actin (h) Fast myosin heavy chains in the TA muscle of 15 week old *Nr1d1*^*-/-*^ mice (n = 3). *p<0.05 and **p<0.01 were determined by a One-Way ANOVA. RT-qPCR data are expressed as mean ± s.e.m.

To investigate why 15 weeks old *Nr1d1*^*+/-*^ and *Nr1d1*^*-/-*^ mice displayed varying lean mass levels we analyzed markers of cellular stress and protein metabolism. *Nr1d1*^*+/-*^ mice displayed no variation in gene expression of cellular stress markers *Ddit4*, *Map3k6*, *Map3k8*, *Map3k14*, *Tgif1*, *Mknk2*, *Junb*, and *Cebpb* (S3A Fig*)*. Ddit4 (or Redd1) is activated by DNA damage and inhibits mTOR signaling in SkM [24]. Interestingly the expression of the cell growth inhibitor *Ddit4* actually decreased in the *Nr1d1*^*+/-*^ mice. We also found no global differences in catabolic and anabolic gene expression between *Nr1d*^*+/-*^ and Nr1d1^+/+^ mice although *Nr1d1*^*+/-*^ mice displayed enhanced expression of *Smad2* and *p38Mapk* and *Akt2* (Fig 3A–3D). However, when *Nr1d1* expression was completely lost, catabolic gene expression and cellular stress expression increased significantly (Fig 3A and 3B and S3B Fig). There was also a mild average increase in cleaved Caspase 3 levels in Rev-erbα-null mouse tibialis anterior muscle, indicating enhanced protease activity (S3C Fig). *Rev-erbα* null mice showed elevated catabolic gene expression (*Tgf-b*, *Foxo3a*, *Smad2* and *p38Mapk*) (Fig 3A and 3B) with a concomitant reduction in anabolic gene expression (Fig 3C and 3D), in agreement with previous findings [19]. Further investigation revealed an increase in FOXO3A protein expression (Fig 3G) and STAT3 Ty705 phosphorylation known to be associated with enhanced muscle degradation were more highly activated in the SkM of the *Nr1d1*^*-/-*^ mice (Fig 3G). Interestingly, although *Akt* expression was elevated in *Nr1d1*^*+/-*^ compared to wildtype and knockout mice, this effect was not conserved at the protein level (Fig 3G). In concordance with these findings, we detected a decrease in oxidative (slow) myosin heavy chain 1A (MHC1A) (Fig 3G) and glycolytic (fast) myosin heavy chain 2B (MHC2B) (Fig 3H) in the SkM of the *Nr1d1*^*-/-*^ mice compared to other genotypes. In the highly oxidative soleus muscle, a muscle fiber known to show strong phenotypic stratification in response to loss of *Nr1d1*, heterozygosity partly induced glucose metabolism without influencing fatty acid oxidation (S4A–S4D Fig). Overall, these data support the idea that *Rev-erbα* influences SkM protein stability through suppression of catabolic pathways particularly in older mice; a process that is disrupted in *Nr1d1*-null mice.

### Decreased *Rev-erbα* expression induces fatty acid oxidation in mice

**Fig 4:**
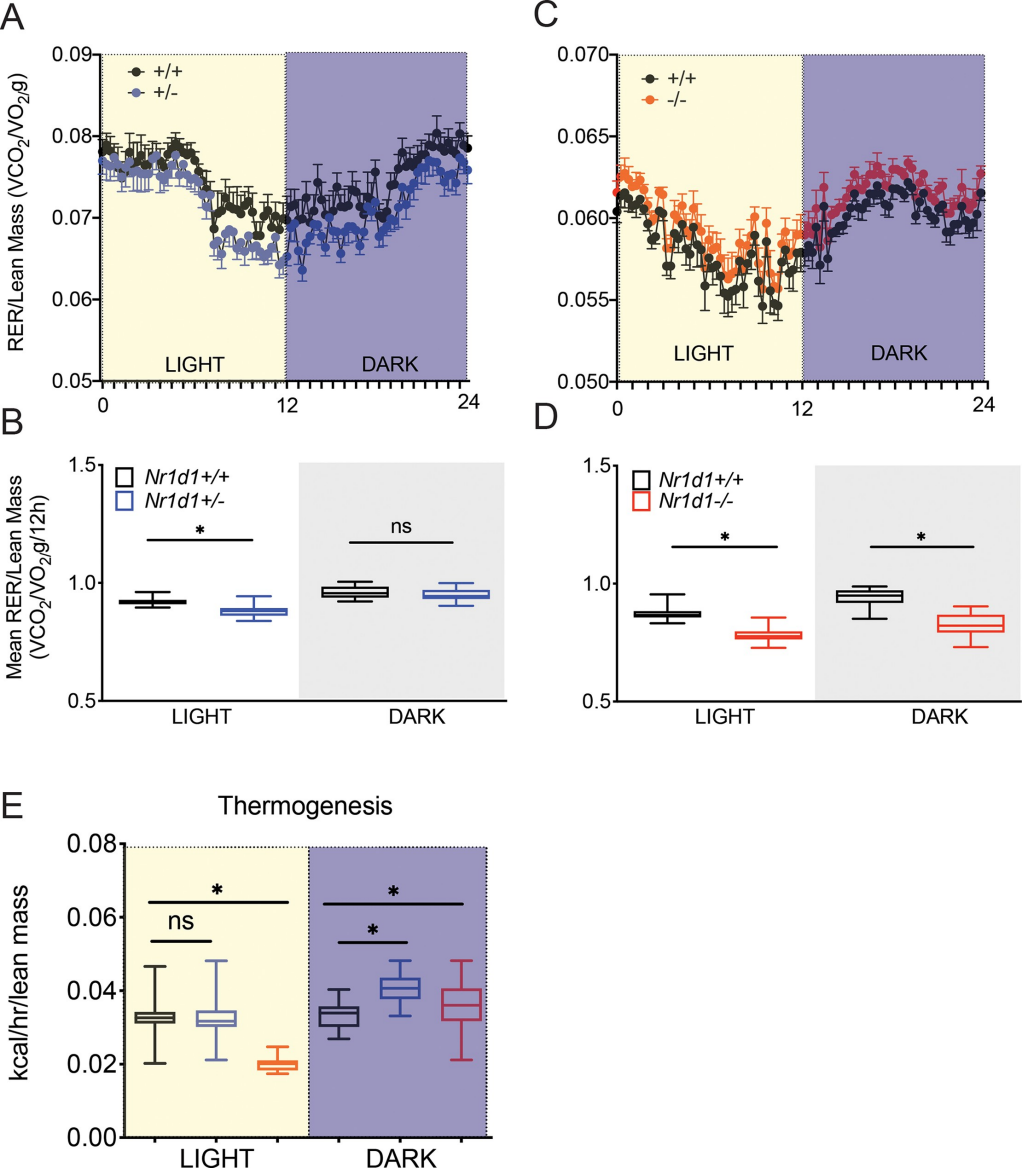
Reduced *Nr1d1* expression modulates diurnal fatty acid utilization. (a) Metabolic trace showing diurnal changes in the Respiratory Exchange Ratio (RER:VO2/VCO2 per unit mass in g) in *Nr1d1*^*+/+*^ and *Nr1d1*^*+/-*^ mice (b) Mean RER for each genotype over a 12 h light or dark period (a) (c) 24 hr day/night analysis showing diurnal changes in the RER of *Nr1d1*^*+/+*^ and *Nr1d1*^*-/-*^ mice (d) Mean RER for each genotype over the time period shown in (b). All mice were kept on a 12:12 light/dark cycle at room temperature (n = 7). Whole body metabolism and thermogenesis was quantified using a Comprehensive Lab Animal Monitoring System (CLAMS). VO2 and VCO2 and RER were normalized to the lean mass of the animals. *p<0.05 was determined by a student’s t-test where relevant or One-Way ANOVA. Data represented as box and whiskers plot error bars +/- stdev.

REV-ERBα is a regulator of metabolism and thermogenesis [25, 26]. However, whether *Nr1d1*^*+/-*^ mice exhibit a difference in whole body metabolism has not been previously elucidated. Adult *Nr1d1*^*+/-*^ mice, 12–14 weeks old, were placed in metabolic cages to quantify whole-body metabolism, using indirect calorimetry, and heat production. As expected, NMR-analysis revealed that *Nr1d1*^*+/-*^ mice had higher total body weight due to higher fat mass and increased lean mass relative to wildtype mice (S5A–S5C Fig). Interestingly, we found a minor drop in the respiratory exchange ratio (RER) in heterozygous mice to a value of 0.88 during the light cycle, representing a modest increase in fatty acid substrate preference (Fig 4A and 4B). As reported previously, knockout animals displayed a lower RER during a 24h period (Fig 4C and 4D). However, we found no significant difference in the substrate preference during the dark cycle in *Nr1d1*^*+/-*^ and *Nr1d1*^*+/+*^ mice (Fig 4A and 4B). Lastly, *Nr1d1*^*+/-*^ mice displayed no changes in body heat production during the light cycle (Fig 4E). Still, during the dark cycle the Nr1d1-heterozygosity led to a mild increase in heat production compared to wildtype and knockout mice during the dark cycle (Fig 4E). These data suggest that even partially reducing *Rev-erbα* expression is sufficient to augment fatty acid metabolism and thermoregulation in a circadian dependent manner.

### *Rev-erbα* heterozygosity expression increases glucose clearance independent of lean mass

**Fig 5:**
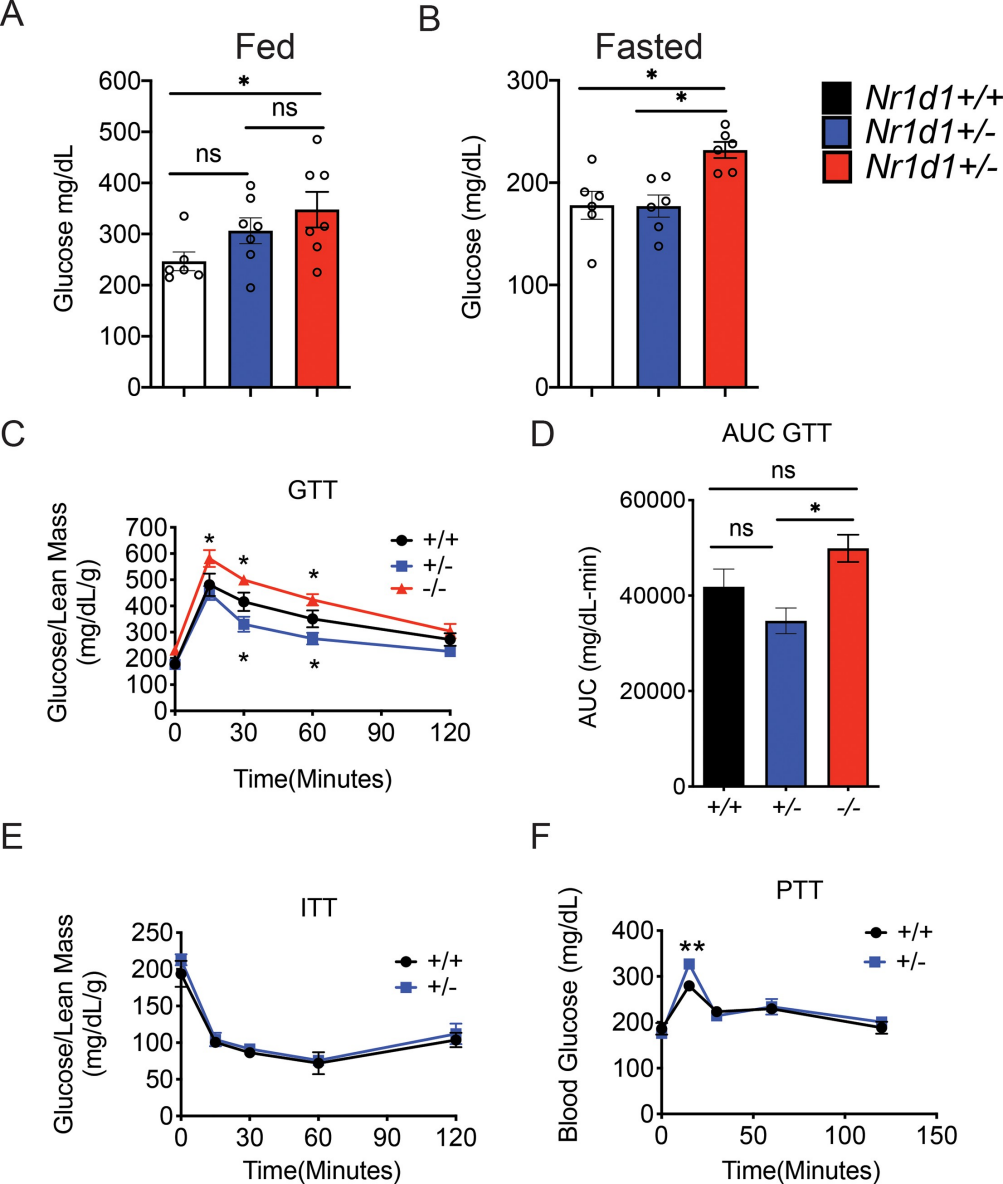
Partial loss of the *Nr1d1* gene promotes glucose clearance independent of lean mass levels. (a) Blood glucose levels in fed mice. (b) Blood glucose levels in mice fasted for 4 h. (c) Glucose tolerance and (d) area under the curve analysis (AUC) of *Nr1d1*^*+/+*^, *Nr1d1*^*+/-*^ and *Nr1d1*^*-/-*^ mice. (e) Insulin and (f) pyruvate tolerance in *Nr1d1*^*+/+*^ and *Nr1d1*^*+/-*^ mice. Mice where age matched male littermates 10–12 weeks of age when conducting GTT, 11–13 weeks of age when conducting the ITT, and 12–14 weeks of age when conducting the PTT (n = 6). GTT and ITT were normalized to the lean mass of the animals. *p<0.05 and **p<0.01 were determined by One-Way ANOVA or Two-Way ANOVA where relevant. Data are expressed as mean ± s.e.m.

To further characterize the metabolic effects of partial *Nr1d1* expression we decided to determine if wildtype and heterozygous mice displayed differences in glucose uptake, insulin sensitivity, and gluconeogenesis. To achieve this goal, we conducted a glucose tolerance test (GTT), an insulin tolerance test (ITT), and a pyruvate tolerance test (PTT) on *Nr1d1*^*+/-*^ and *Nr1d1*^*+/+*^ mice. We found no significant differences in the pre- and post-fast body weights of *Nr1d1*^*+/-*^ and *Nr1d1*^*+/+*^ mice (S6A Fig). Importantly, heterozygous mice retained a higher percentage of lean mass and a similar percentage of fat mass to pre-fast wildtype mice (S6A–S6C Fig). Nr1d1^+/-^ mice showed blood glucose levels equivalent to that of wildtype mice whether fed or fasted for 4 hours (Fig 5A and 5B). *Nr1d1-*heterozygous mice also displayed enhanced glucose clearance compared to wildtype mice (Fig 5C and 5D). However, heterozygous mice did not display any differences in insulin sensitivity compared to wildtype mice, as revealed by an insulin tolerance test (ITT) (Fig 5E). It should be noted that *Nr1d1*^*-/-*^ mice have not been shown to exhibit differences in glucose clearance and insulin sensitivity compared to wildtype mice, but do exhibit 10% higher resting blood glucose levels. Our observations were consistent with these previous findings (Fig 5C–5E) [14]. We also found that partial reduction of *Rev-erbα* expression mildly increased gluconeogenesis as heterozygous mice exhibit a short-lived increase in blood glucose levels in response to a bolus of pyruvate (Fig 5F). These results highlight the nuanced role of REV-ERBα in the regulation of glucose homeostasis and affirms the role of REV-ERBα as a mediator of gluconeogenesis in *vivo*.

### Heterozygous *Rev-erbα* mice display a unique gene expression profile in distinct metabolic tissues

The partial reduction of *Rev-erbα* expression in *Nr1d1*^*+/-*^ mice induced a change in fatty acid oxidation and glucose metabolism that is not mirrored in *Rev-erbα* null mice [21, 27]. To gain a further understanding of the unique metabolic phenotype of *Nr1d1*^*+/-*^ mice, we probed whether partial *Rev-erbα* expression differentially modulated glycolytic and fatty acid oxidation in epididymal white adipose tissue (eWAT), brown adipose tissue (BAT), the liver, and soleus muscle. In the soleus muscle, we observed that *Rev-erbα* null mice showed an increase in fatty acid oxidation enzymes with a mild increase in expression of the glycolytic enzyme hexokinase 1 (*Hk1*) of the direct Rev-Erb target genes surveyed (S6A Fig, S6C Fig). Conversely, heterozygous mice displayed no differences in FAO gene expression (S6B Fig), but exhibited an analogously mild decrease in the glycolytic genes *Hk1* and *Hk2* (S6D Fig). These results highlighted that glycolytic and fatty-acid oxidation may not be differentially regulated in response to partial loss of Rev-Erbα.

**Fig 6:**
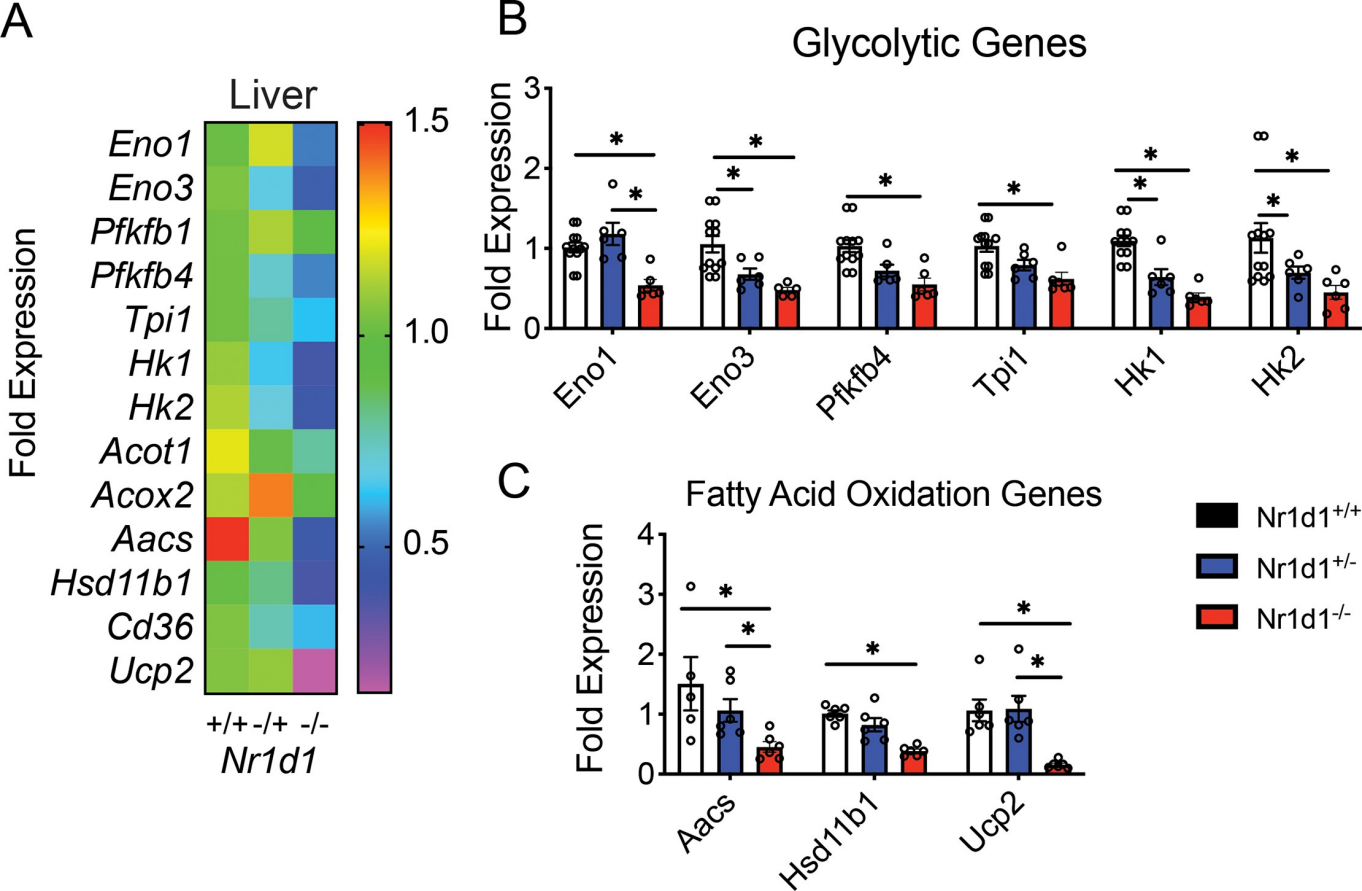
Heterozygous Nr1d1 mice display a reduction in glycolytic gene expression and preserved fatty acid metabolism in the liver. (a) Heat-map showing RT-qPCR-quantified expression pattern of glycolytic (*Eno1*, *Eno3*, *Pfkfbp1*, *Pfkfbp4*, *Tpi1*, and fatty-acid oxidation enzymes (*Hk1*, *Hk2*, *Acot1*, *Acox2*, *Aacs*, *Hsb11b1*, *Cd35*, *Ucp1*) in the livers *Nr1d1* ^*+/-*^, *Nr1d1*^*+/-*^ and *Nr1d1*^*-/-*^ mice (b) Mean glycolytic gene expression in the livers of 15-week-old *Nr1d1* ^*+/-*^, *Nr1d1*^*+/-*^ and *Nr1d1*^*-/-*^ mice (n = 5). (c) *p<0.05 determined by One-Way ANOVA. Data represented as a mean ± s.e.m.

In *Nr1d*^*-/-*^ mice livers RT-qPCR analysis revealed a drastic decrease in glycolytic enzyme expression with also a decrease in fatty acid oxidation genes (Fig 6A and 6B). Intriguingly, Nr1d1^+/-^ liver showed a pronounced reduction in the expression of the glycolytic genes *Eno3*, *Hk1* and *Hk2* (Fig 6B and 6C), suggesting that *Nr1d1* heterozygosity may influence hepatic glucose metabolism. Conversely, heterozygous mice did not show differential regulation of FAO enzyme expression compared to wildtype mice (Fig 6C).

In BAT, Nr1d1^-/-^ mice uniquely exhibited a significant reduction in the glycolytic enzymes *Pfk1* and an induction of enolase 1 (*Eno1*) and triosphosphate-isomerase 1 (*Tpi1*) with partial *Rev-erbα* loss having no effect on these genes (S7A Fig). Expression of *Hk1* and *Hk2*, however were similarly upregulated in both genotypes (S7A Fig). Intriguingly only *Upc1* was upregulated in *Nr1d1*^*-/-*^ mice relative to wildtype and heterozygous mice, whereas other FAO factors such as *Ucp2*, *Ucp3* and *Cpt1a* being similarly downregulated in heterozygous and knockout mice versus wildtype mice (S7B Fig). It is known that Rev-erbα null mice exhibit an elevated heat expenditure through increased *Ucp1* activity in BAT [28]. However, these results also highlighted that Rev-Erbα suppresses *Ucp2* and *Ucp3* gene expression in BAT.

**Fig 7:**
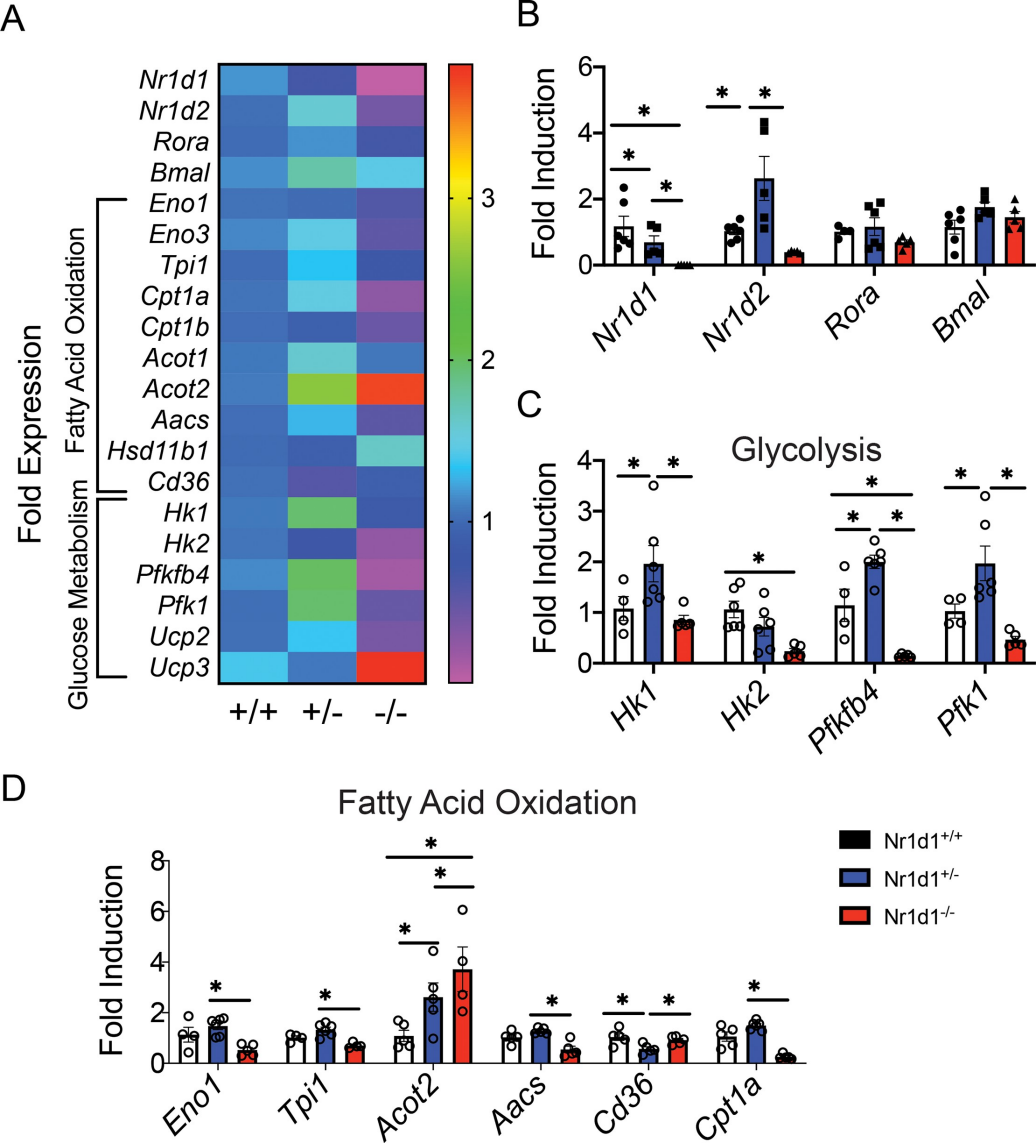
Heterozygous *Nr1d1* mice exhibit enhanced metabolic gene expression in epididymal in white adipose tissue (eWAT). (a) Heatmap showing expression of glycolytic and fatty acid oxidation genes in the eWAT of 15-week-old *Nr1d1*^*+/+*^ *Nr1d1*^*+/-*^ and *Nr1d1*^*-/-*^ mice. (b) Mean expression of *Nr1d1*, *Nr1d2* and *Rora* and *Bmal 1* in eWAT. (c) Mean expression of the glycolytic enzymes *Hk1*, *Hk2*, *Pfkfbp4* and *Pfk1*. (d) Gene expression was quantified using RT-qPCR. *p<0.05 and **p<0.01 were determined by One-Way AOVA. Data represented as a mean ± s.e.m (n = 6).

In order to elucidate differences in Rev-Erbα mediated regulation of white adipose tissue metabolism, we also profiled glycolysis and FAO gene expression in WAT of *Nr1d1*^*-/-*^ and *Nr1d1*^*+/-*^ mice. We found that *Rev-erbα* produced an inverse expression pattern for FAO and glycolytic genes depending on the level of gene expression. In *Nr1d1* null mice eWAT displayed a decrease in glycolytic (*Pfk1*, *Pfkfbp4*, *Hk1 and Tpi1)* and FAO expression (*Ucp2*, *Aacs* and *Cpt1a*) yet these factors were elevated in *Nr1d1*^*+/-*^ mice (Fig 7A–7D). The fatty acid transporter *Cd36* was the only FAO factor downregulated in Nr1d1+/- mice compared to wildtype of knockouts (Fig 7A and 7D). These data suggest that decreased Rev-erbα expression stimulated, while in contrast complete loss of *Nr1d* paradoxically repressed eWAT metabolism. Suggesting the differences in metabolic phenotypes between the heterozygous and null mice may be due to a dose dependent differential regulation of glycolytic and fatty acid gene expression in WAT tissue.

## Discussion

Circadian proteins are key regulators of metabolism and dictate susceptibility to metabolic syndromes [6, 11, 14, 27]. The nuclear receptor REV-ERBα is a key repressor of the molecular clock and modulates metabolic and developmental pathways. Our study wished to thoroughly understand how *Rev-erbα* expression levels impacts metabolism and muscle morphology due to a previous observation that *Nr1d1* heterozygous mice have superior muscle fiber size [16]. In this study we illustrate that *Nr1d1*-heterozygosity increased glucose clearance, fatty acid utilization, gluconeogenesis, and also boosted lean mass accumulation without generating higher adiposity in chow-fed mice.

Distinct differences in phenotype has been rarely reported with a beneficial effect of heterozygosity when compared to null animals. For example, this “Goldilocks Effect” has been described in *Ctrf* mice resulting in decreased compliance and increased resistance in the lung and effectively mirroring the cystic fibrosis [29]. However, when mice were heterozygous for the mutated *Ctrf* allele it did not induce a classical gene dosage effect that was predicted by the phenotype. Surprisingly, *Ctrf*^*+/-*^ mice displayed decreased resistance and enhanced compliancy within the lung [29]. Resulting in a heterozygous advantage over both the null and wild type animals. This study, however, is the first to highlight the underlying contributory factors that drive the discordance between *Nr1d1* heterozygous and knockout mice. Our investigations aver that complete loss of REV-ERBα has deleterious effects on muscle homeostasis through enhanced metabolism of fatty acids in muscle and depressed glucose utilization and FAO in eWAT. Conversely, heterozygous mice displayed a bias toward fatty acid oxidation in eWAT during inactive periods, possibly driven by enhanced glycolytic and FAO enzyme expression. Heterozygous mice also interestingly exhibited enhanced thermogenesis during times of activity and enhanced glucose and pyruvate tolerance. Most importantly we show that the negative effects of REV-ERBα loss in muscle may in part be driven by disregulated muscle catabolism via FOXO3A and Stat3 activation in an age-dependent manner.

The mechanism(s) through which *Nr1d1* heterozygosity boosts lean mass levels at an early age is not clear. Our data demonstrates that *Nr1d1* null mice display significant gains in fat mass with a concomitant increase in muscular atrophy and an associated loss in muscle function. Intriguingly, heterozygous *Nr1d1* mice exhibit no changes in adiposity, showed increased grip strength, and enhanced muscle mass without significant changes in the expression of anabolic or catabolic factors in the skeletal muscle. Myogenic programs are highly active during neonatal growth and are one of the primary drivers in the development of the musculature [30]. We have found that myogenic gene expression is enhanced in *Nr1d1* heterozygous mice only when subjected to skeletal muscle injury, suggesting the *Nr1d1* heterozygosity is beneficial in skeletal muscle only under a promyogenic environment [16]. In mature post mitotic skeletal muscle we found no differences in metabolic gene expression among heterozygous versus wiltype mice, implying that reduced Rev-erbα expression may not impact mature skeletal muscle energetics. Therefore, it is plausible that mature *Nr1d1*^*+/-*^ mice experience the benefits of increased lean mass and strength due to key events early on in muscle development that facilitates and exponentially amplified increase in muscle mass by full maturity. *Rev-erbβ* is also highly expressed in post mitotic skeletal muscle and in some cases can act as an axillary component to REV-ERBα-mediated gene repression [23, 31]. Both *Rev-erbα* and *Rev-erbβ* expression is drastically reduced during myoblast differentiation [32, 33]. This study suggests that there may be an ideal threshold at which REV-ERBα expression facilitates elevated fatty oxidation in eWAT and lean mass accumulation. Amador and colleagues have suggested that REV-ERBβ in contrast to REV-ERBα knockouts may also exhibit muscle hypertrophy through enhanced food intake [18]. Here we show that partial loss of Rev-erbα expression alone sufficiently enhances lean mass without influence feeding behavior or activity. Importantly, differences between the *Rev-erbα* heterozygote and wildtype phenotype have only been assessed in skeletal muscle undergoing developmental growth or regenerative repair. Whether partial loss recapitulates the deleterious effects of Rev-erbα ablation on endurance and mitochondrial function should be the subject of future investigations.

Collectively our results indicate that Nr1d1 heterozygous mice exhibit a drastic departure from the phenotype of knockout mice. The data indicates that partial loss of *Nr1d1* gene does not produce deleterious effects and effectively augments metabolic substrate preference. This aligns well with our previous studies in which we showed that REV-ERBα inhibition is an effective approach for pharmacologically stimulating muscle repair [16, 17].

Polymorphisms in the *NR1D1* gene has been associated with obesity in various human populations [34, 35]. To our knowledge a correlation between lean mass and REV-ERBα SNPs has not been reported. New studies investigations that venture beyond assessments of fat mass levels and designed to probe the effect of *NR1D1* SNPs on whole-body metabolism may further shed light on the role of *REV-ERBα* expression in metabolic regulation in humans.

## Supporting information

S1 Fig Heterozygous gene expression of *Nr1d1* does not cause the disruption of circadian activity. (a) Actogram showing representing wheel-running activity in 6–8 weeks old *Nr1d1*^*+/+*^and *Nr1d1*^*+/-*^ mice. Mice were kept in circadian cabinets on a strict 12h/12h Light/Dark cycle. (b) Actograms showing wheel-running activity of 7–9 weeks old *Nr1d1*^*+/-*^ and *Nr1d1*^*+/+*^ mice exposed to continual darkness. Mice were kept in circadian cabinets on a strict 24h dark cycle. Black arrows indicate earliest signs of shift in circadian behavior that is conserved across both genotypes. Actograms were generated using Matlab Clocklab.
(PDF)

S2 Fig Heterozygous gene expression of *Nr1d1* does not disrupt feeding behavior or intake. (a) Daily food intake, (b) daily water intake, (c) and total feeding events per day for wild type *Nr1d1*^*+/+*^ and *Nr1d1*^*+/-*^ mice (n = 6). Food intake on 8–10 week-old *Nr1d1*^*+/+*^ and *Nr1d1*^*+/-*^ mice was assessed over 14 days using the BioDAQ episodic intake monitor. Mice were allowed food and water *ad libitum*. Data was collected and analyzed using BioDaq software. Data are expressed as mean ± s.e.m. No statistical significance was found between the two groups by using a student t-test.
(PDF)

S3 Fig The loss of *Nr1d1* gene promotes cellular stress in the skeletal muscle. (a-b) Expression of cellular stress genes in the soleus muscle of 15 week old *Nr1d1*^*+/-*^ and *Nr1d1*^*-/-*^ mice (n = 6). Gene expression determined by RT-qPCR. (c) Immunoblot analysis of (c) Caspase 3 in the quadricep muscle of 15-week-old *Nr1d1*^*-/-*^ mice (n = 3). *p<0.05 and **p<0.01 were determined by One-Way ANOVA. Data are expressed as mean ± s.e.m.
(PDF)

S4 Fig Soleus muscle from heterozygous mice displays normal metabolic gene expression when compared to wildtype mice. (a) Heatmap showing the pattern of expression of fatty acid oxidation and glucose metabolism enzymes in the soleus muscle of 15-week-old *Nr1d1*^*+/+*^ *Nr1d1*^*+/-*^ and *Nr1d1*^*-/-*^ mice (c-d) glycolytic gene expression in the soleus muscle of 15 weeks old *Nr1d1*^*-/-*^ and *Nr1d1*^*+/-*^ mice (n = 6). Mean expression of *Nr1d1*, *Nr1d2* and *Rora* and *Bmal 1* in soleus muscle. (c) Mean expression of the fatty acid oxidation genes *Eno1 and Tpi1*. (d) Average gene expression of the glucose metabolism factors *Cpt1a*, *Cpt1b*, *Hk1*, *Hk2*, *Pfk1*, *Ucp1*, *Ucp2* and *Ucp3*. Gene expression quantified using RT-qPCR. *p<0.05 and **p<0.01 were determined by One-Way ANOVA. Data represented as a mean ± s.e.m (n = 6).
(PDF)

S5 Fig Body composition of *Nr1d1*^*+/-*^ mice placed into metabolic cages. All mice were kept on a 12:12 light/dark cycle at room temperature (n = 6). (a) Total, (b) fat and (c) lean mass of *Nr1d1*^*+/+*^ and *Nr1d1*^*+/-*^ mice. Fat and lean mass were determined by using a Bruker BioSpin LF50 Body Composition Analyzer before placing mice into metabolic cages. *p<0.05 and **p<0.01 were determined by One. Data are expressed as mean ± s.e.m.
(PDF)

S6 Fig Representative body composition data of *Nr1d1*^*+/-*^ mice before metabolic tolerance tests. (a) Pre-Fast and Post-Fast body weights of *Nr1d1*^*+/+*^ *and Nr1d1*^*+/-*^ mice (n = 6). (b) Percent lean and (c) fat mass were determined by using a Bruker BioSpin LF50 Body Composition Analyzer before each fast. *p<0.05 was determined by two tailed-student’s t-test. Data are expressed as mean ± s.e.m.
(PDF)

S7 Fig Rev-Erb heterozygosity modulates metabolic gene expression in brown adipose tissue (BAT). (a) Mean expression of the glycolytic factors *Pfk1*, *Eno1*, *Hk1*, *Hk2*, *Tpi1* (b) Mean expression of the fatty acid oxidation factors *Cpt1a*, *Cpt1b*, *Ucp1*, *Ucp2* and *Ucp3*. mRNA was isolated from BAT using Trizol extraction from 15-week-old *Nr1d1*^*+/+*^, *Nr1d1*^*-/-*^ and *Nr1d1*^*+/-*^ mice (n = 6). Gene expression was determined by RT-qPCR. *p<0.05 and **p<0.01 were determined by One-Way ANOVA. Data represented as a mean ± s.e.m.
(PDF)
