# Supplementary material for: Rev-erbα heterozygosity produces a dose-dependent phenotypic advantage in mice
Source: PLoS One. 2020 May 14;15(5):e0227720. doi: 10.1371/journal.pone.0227720 (PMC7224546; doi:10.1371/journal.pone.0227720)
Supplement: S1 Fig — (a) Actogram showing representing wheel-running activity in 6–8 weeks old Nr1d1+/+and Nr1d1+/- mice. Mice were kept in circadian cabinets on a strict 12h/12h Light/Dark cycle. (b) Actograms showing wheel-running activity of 7–9 weeks old Nr1d1+/- and Nr1d1+/+ mice exposed to continual darkness. Mice were kept in circadian cabinets on a strict 24h dark cycle. Black arrows indicate earliest signs of shift in circadian behavior that is conserved across both genotypes. Actograms were generated using Matlab Clocklab. (PDF) [file pone.0227720.s001.pdf]

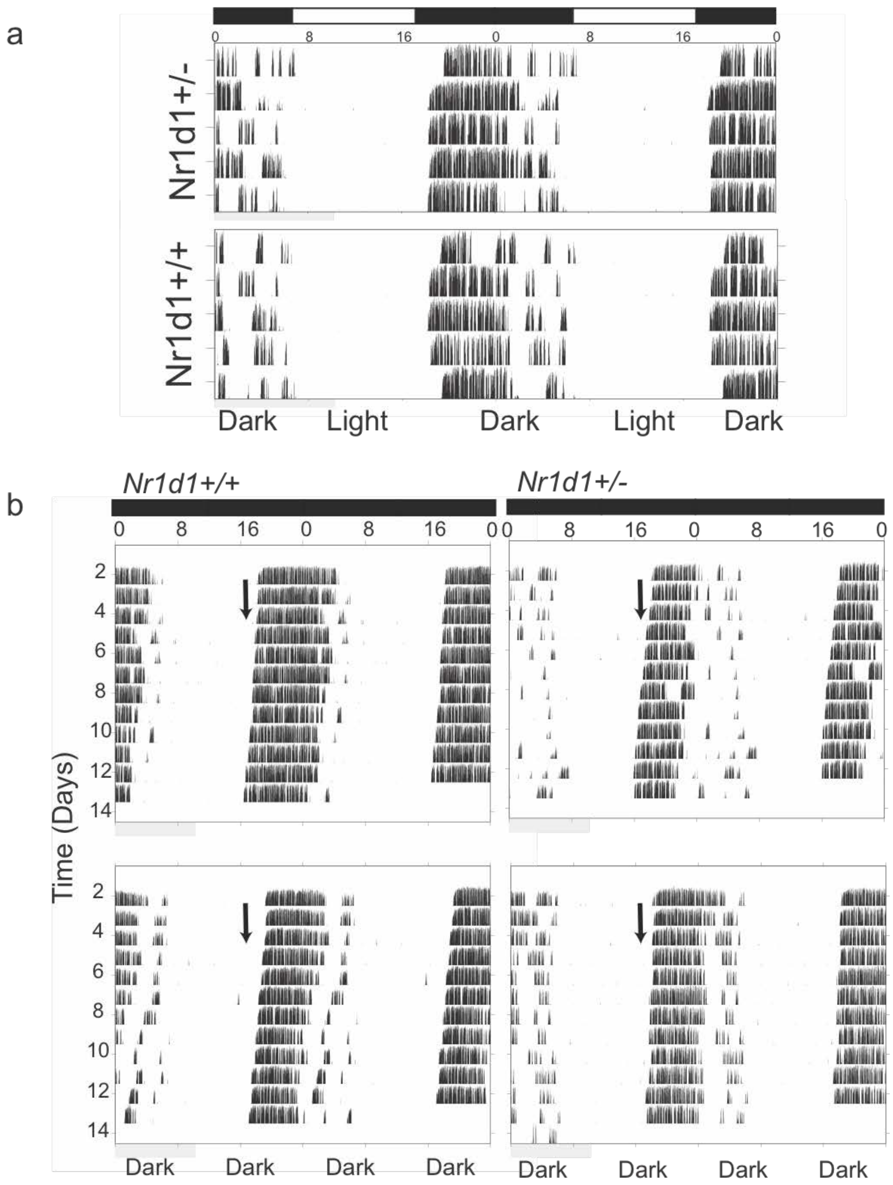

Supplemental Fig S1. Heterozygous gene expression of *Nr1d1* does not cause the disruption of circadian activity. (a) Actogram showing representing wheel-running activity in 6-8 weeks old *Nr1d1*$^{+/+}$and *Nr1d1*$^{+/-}$ mice. Mice were kept in circadian cabinets on a strict 12h/12h Light/Dark cycle. **(b)** Actograms showing wheel-running activity of 7-9 weeks old *Nr1d1*$^{+/-}$ and *Nr1d1*$^{+/+}$ mice exposed to continual darkness. Mice were kept in circadian cabinets on a strict 24h dark cycle. Black arrows indicate earliest signs of shift in circadian behavior that is conserved across both genotypes. Actograms were generated using Matlab Clocklab.
