# Supplementary material for: Rev-erbα heterozygosity produces a dose-dependent phenotypic advantage in mice
Source: PLoS One. 2020 May 14;15(5):e0227720. doi: 10.1371/journal.pone.0227720 (PMC7224546; doi:10.1371/journal.pone.0227720)
Supplement: S2 Fig — (a) Daily food intake, (b) daily water intake, (c) and total feeding events per day for wild type Nr1d1+/+ and Nr1d1+/- mice (n = 6). Food intake on 8–10 week-old Nr1d1+/+ and Nr1d1+/- mice was assessed over 14 days using the BioDAQ episodic intake monitor. Mice were allowed food and water ad libitum. Data was collected and analyzed using BioDaq software. Data are expressed as mean ± s.e.m. No statistical significance was found between the two groups by using a student t-test. (PDF) [file pone.0227720.s002.pdf]

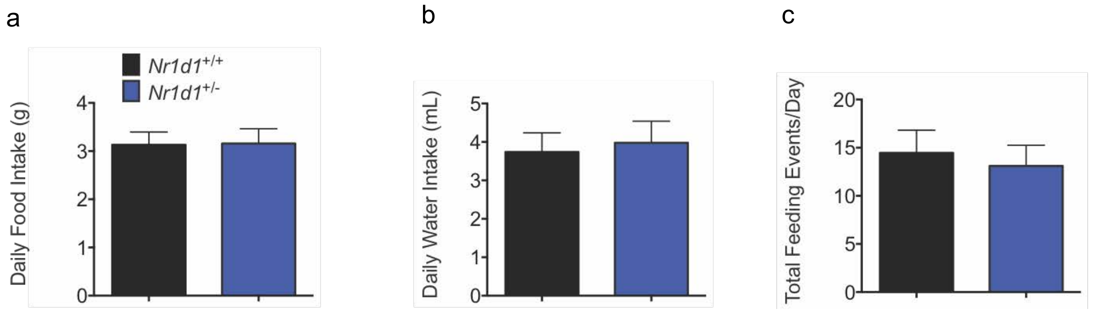

Supplemental Fig S2. Heterozygous gene expression of *Nr1d1* does not disrupt feeding behavior or intake. (a) Daily food intake, **(b)** daily water intake, **(c)** and total feeding events per day for wild type *Nr1d1*$^{+/+}$ and *Nr1d1*$^{+/-}$ mice (n = 6). Food intake on 8-10 week-old *Nr1d1*$^{+/+}$ and *Nr1d1*$^{+/-}$ mice was assessed over 14 days using the BioDAQ episodic intake monitor. Mice were allowed food and water *ad libitum*. Data was collected and analyzed using BioDaq software. Data are expressed as mean ± s.e.m. No statistical significance was found between the two groups by using a student t-test.
