# Supplementary material for: Rev-erbα heterozygosity produces a dose-dependent phenotypic advantage in mice
Source: PLoS One. 2020 May 14;15(5):e0227720. doi: 10.1371/journal.pone.0227720 (PMC7224546; doi:10.1371/journal.pone.0227720)
Supplement: S3 Fig — (a-b) Expression of cellular stress genes in the soleus muscle of 15 week old Nr1d1+/- and Nr1d1-/- mice (n = 6). Gene expression determined by RT-qPCR. (c) Immunoblot analysis of (c) Caspase 3 in the quadricep muscle of 15-week-old Nr1d1-/- mice (n = 3). *p<0.05 and **p<0.01 were determined by One-Way ANOVA. Data are expressed as mean ± s.e.m. (PDF) [file pone.0227720.s003.pdf]

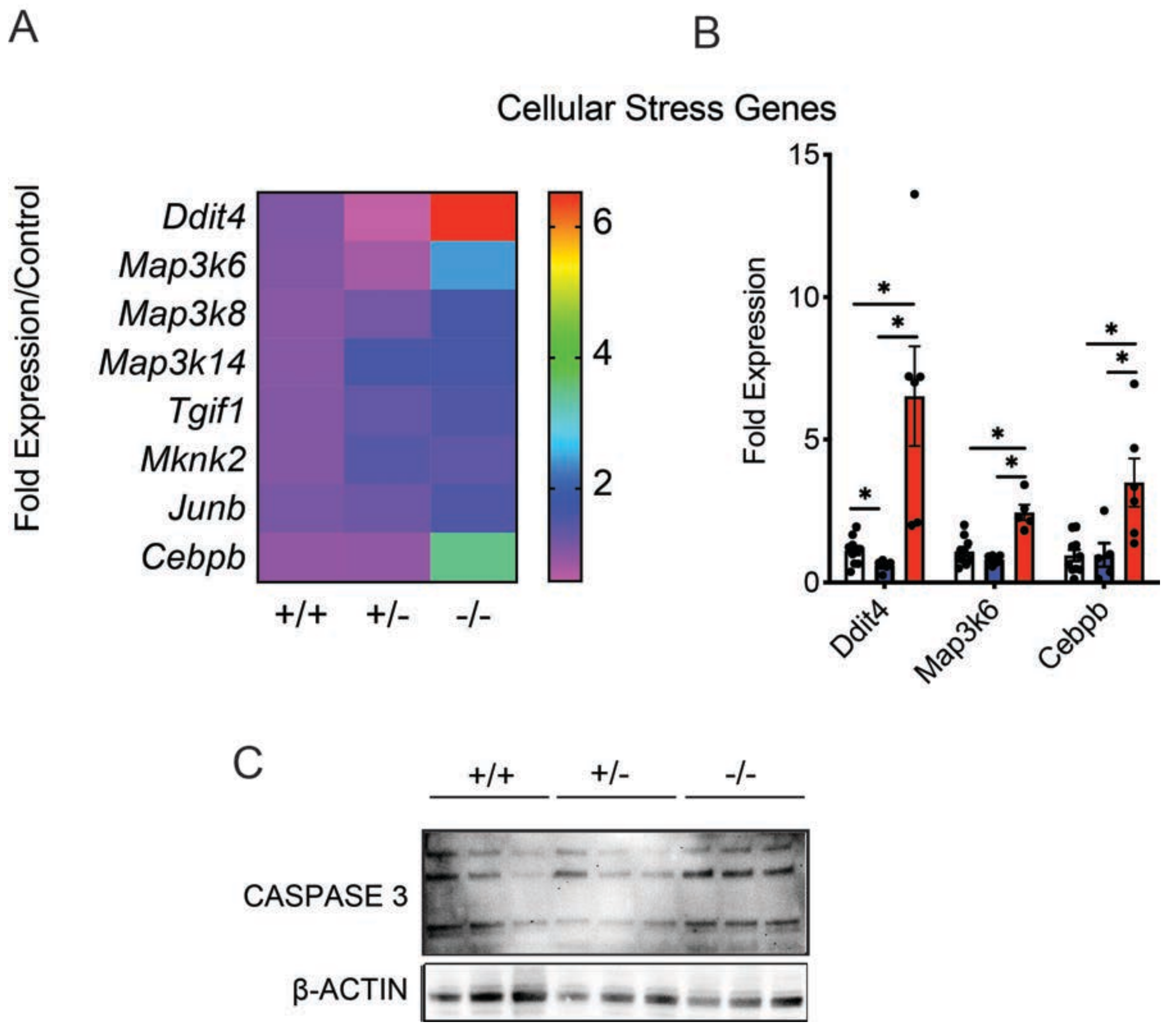

Supplemental Fig S3. The loss of *Nr1d1* gene promotes cellular stress in the skeletal muscle. (a-b) Expression of cellular stress genes in the soleus muscle of 15 week old *Nr1d1*$^{+/-}$ and *Nr1d1*$^{-/-}$ mice (n = 6). Gene expression determined by RT-qPCR. **(c)** Immunoblot analysis of **(c)** Caspase 3 activation in the quadricep muscle of 15-week-old *Nr1d1*$^{-/-}$ mice (n = 3). $^*p<0.05$ and $^{**}p<0.01$ were determined by One-Way ANOVA. Data are expressed as mean ± s.e.m.
