# Supplementary material for: Rev-erbα heterozygosity produces a dose-dependent phenotypic advantage in mice
Source: PLoS One. 2020 May 14;15(5):e0227720. doi: 10.1371/journal.pone.0227720 (PMC7224546; doi:10.1371/journal.pone.0227720)
Supplement: S4 Fig — (a) Heatmap showing the pattern of expression of fatty acid oxidation and glucose metabolism enzymes in the soleus muscle of 15-week-old Nr1d1+/+ Nr1d1+/- and Nr1d1-/- mice (c-d) glycolytic gene expression in the soleus muscle of 15 weeks old Nr1d1-/- and Nr1d1+/- mice (n = 6). Mean expression of Nr1d1, Nr1d2 and Rora and Bmal 1 in soleus muscle. (c) Mean expression of the fatty acid oxidation genes Eno1 and Tpi1. (d) Average gene expression of the glucose metabolism factors Cpt1a, Cpt1b, Hk1, Hk2, Pfk1, Ucp1, Ucp2 and Ucp3. Gene expression quantified using RT-qPCR. *p<0.05 and **p<0.01 were determined by One-Way ANOVA. Data represented as a mean ± s.e.m (n = 6). (PDF) [file pone.0227720.s004.pdf]

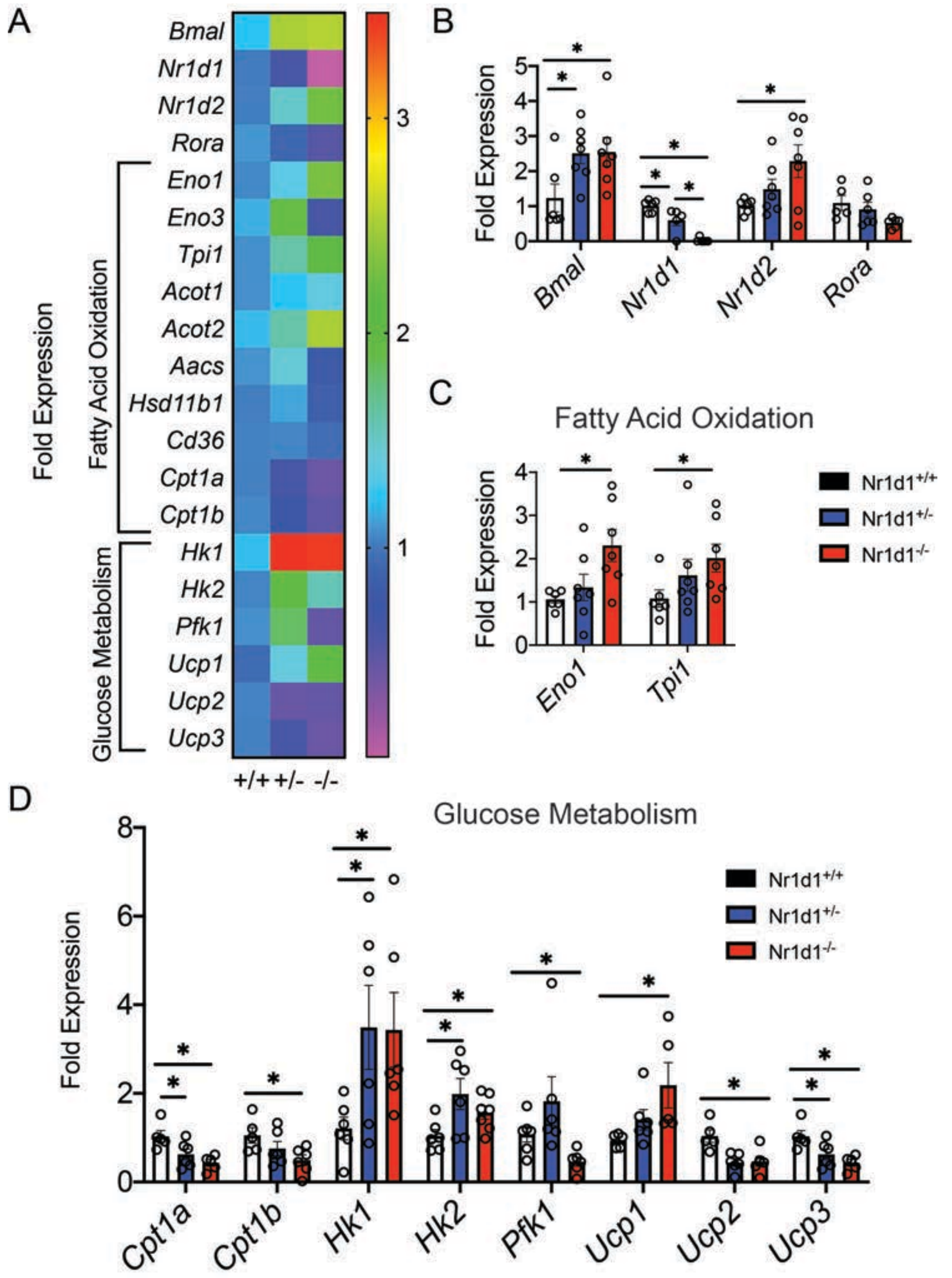

Supplemental Fig S4. Soleus muscle from heterozygous mice displays normal metabolic gene expression when compared to wildtype mice. (a) Heatmap showing the pattern of expression of fatty acid oxidation and glucose metabolism enzymes in the soleus muscle of 15-week-old *Nr1d1*$^{+/+}$ *Nr1d1*$^{+/-}$ and *Nr1d1*$^{-/-}$ mice **(c-d)** glycolytic gene expression in the soleus muscle of 15 weeks old *Nr1d1*$^{-/-}$ and *Nr1d1*$^{+/-}$ mice (n = 6). Mean expression of *Nr1d1*, *Nr1d2* and *Rora* and *Bmal 1* in soleus muscle. **(c)** Mean expression of the fatty acid oxidation genes *Eno1 and Tpi1.* **(d)** Average gene expression of the glucose metabolism factors *Cpt1a, Cpt1b, Hk1, Hk2, Pfk1, Ucp1, Ucp2* and *Ucp3.* Gene expression quantified using RT-qPCR. *p<0.05 and **p<0.01 were determined by One-Way ANOVA. Data represented as a mean ± s.e.m (n = 6)
