# Supplementary material for: Rev-erbα heterozygosity produces a dose-dependent phenotypic advantage in mice
Source: PLoS One. 2020 May 14;15(5):e0227720. doi: 10.1371/journal.pone.0227720 (PMC7224546; doi:10.1371/journal.pone.0227720)
Supplement: S5 Fig — All mice were kept on a 12:12 light/dark cycle at room temperature (n = 6). (a) Total, (b) fat and (c) lean mass of Nr1d1+/+ and Nr1d1+/- mice. Fat and lean mass were determined by using a Bruker BioSpin LF50 Body Composition Analyzer before placing mice into metabolic cages. *p<0.05 and **p<0.01 were determined by One. Data are expressed as mean ± s.e.m. (PDF) [file pone.0227720.s005.pdf]

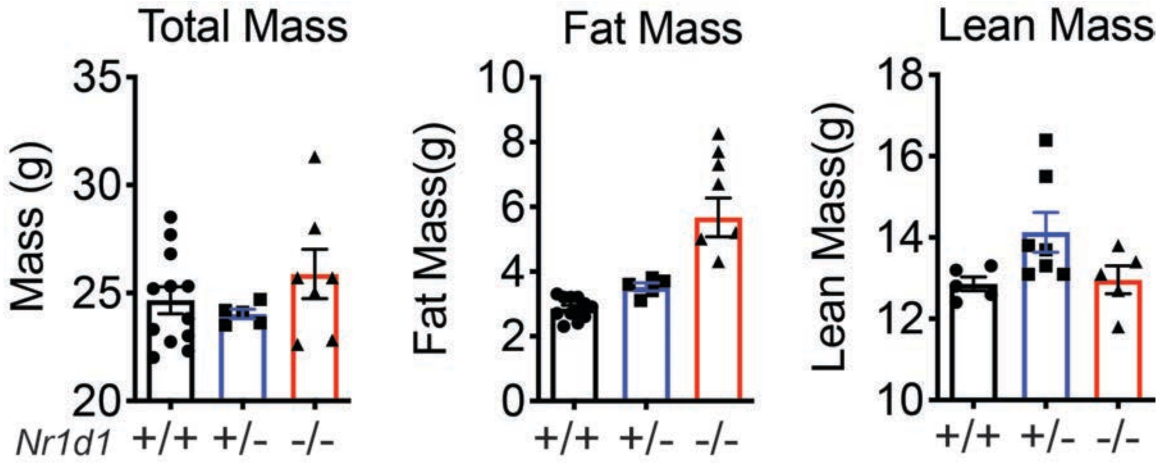

Supplemental Fig S5. Body composition of *Nr1d1*$^{+/-}$ mice placed into metabolic cages. All mice were kept on a 12:12 light/dark cycle at room temperature (n = 6). **(a)** Total, **(b)** fat and **(c)** lean mass of *Nr1d1*$^{+/+}$ and *Nr1d1*$^{+/-}$ mice. Fat and lean mass were determined by using a Bruker BioSpin LF50 Body Composition Analyzer before placing mice into metabolic cages. *p<0.05 and **p<0.01 were determined by One. Data are expressed as mean ± s.e.m.
