# Supplementary material for: Rev-erbα heterozygosity produces a dose-dependent phenotypic advantage in mice
Source: PLoS One. 2020 May 14;15(5):e0227720. doi: 10.1371/journal.pone.0227720 (PMC7224546; doi:10.1371/journal.pone.0227720)
Supplement: S6 Fig — (a) Pre-Fast and Post-Fast body weights of Nr1d1+/+ and Nr1d1+/- mice (n = 6). (b) Percent lean and (c) fat mass were determined by using a Bruker BioSpin LF50 Body Composition Analyzer before each fast. *p<0.05 was determined by two tailed-student’s t-test. Data are expressed as mean ± s.e.m. (PDF) [file pone.0227720.s006.pdf]

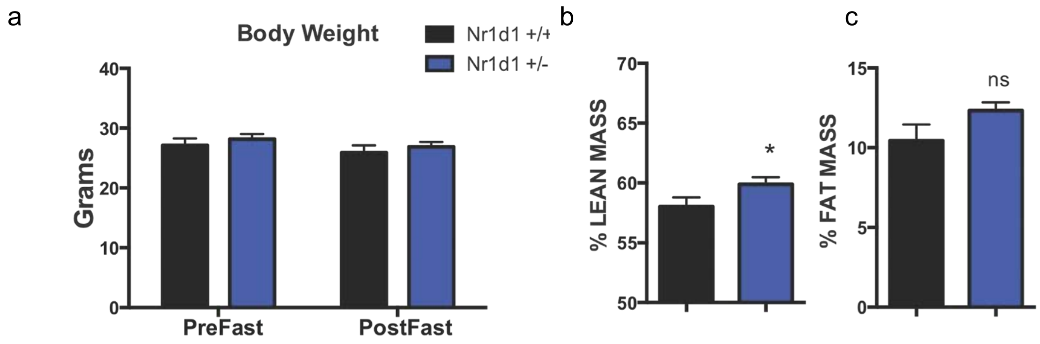

Supplemental Fig S6. Representative body composition data of *Nr1d1*$^{+/-}$ mice before metabolic tolerance tests. (a) Pre-Fast and Post-Fast body weights of *Nr1d1*$^{+/+}$ *and Nr1d1*$^{+/-}$ mice (n =6). **(b)** Percent lean and **(c)** fat mass were determined by using a Bruker BioSpin LF50 Body Composition Analyzer before each fast. *$p<0.05$ was determined by two tailed-student's t-test. Data are expressed as mean ± s.e.m.
