# Supplementary material for: Rev-erbα heterozygosity produces a dose-dependent phenotypic advantage in mice
Source: PLoS One. 2020 May 14;15(5):e0227720. doi: 10.1371/journal.pone.0227720 (PMC7224546; doi:10.1371/journal.pone.0227720)
Supplement: S7 Fig — (a) Mean expression of the glycolytic factors Pfk1, Eno1, Hk1, Hk2, Tpi1 (b) Mean expression of the fatty acid oxidation factors Cpt1a, Cpt1b, Ucp1, Ucp2 and Ucp3. mRNA was isolated from BAT using Trizol extraction from 15-week-old Nr1d1+/+, Nr1d1-/- and Nr1d1+/- mice (n = 6). Gene expression was determined by RT-qPCR. *p<0.05 and **p<0.01 were determined by One-Way ANOVA. Data represented as a mean ± s.e.m. (PDF) [file pone.0227720.s007.pdf]

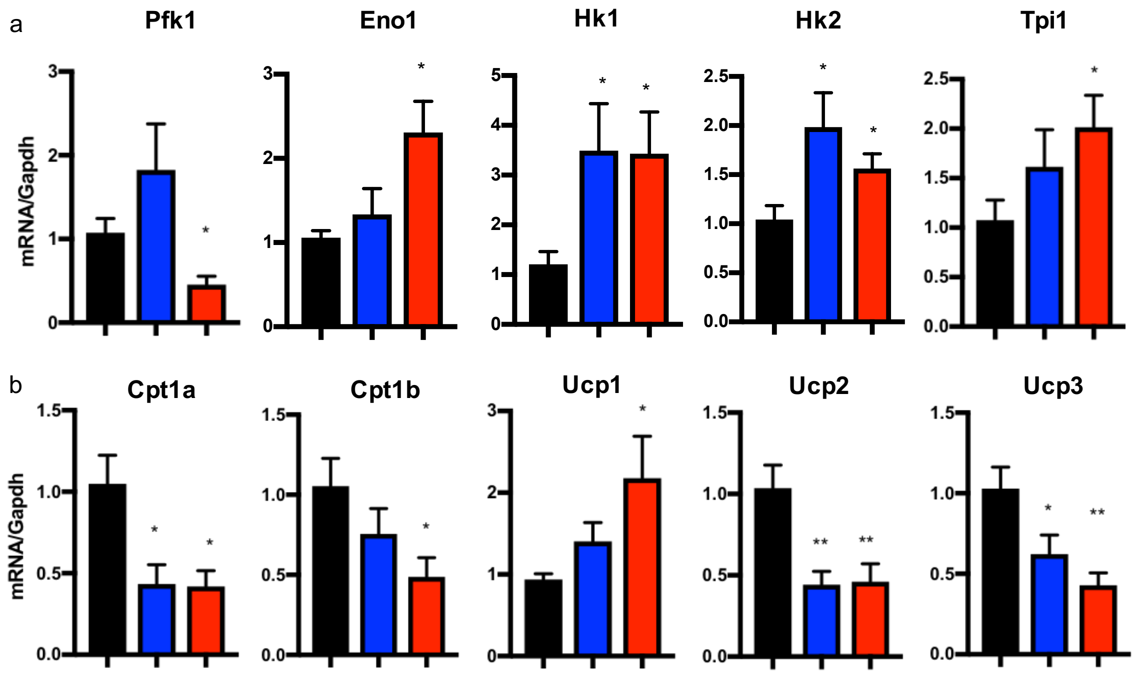

Supplemental Fig S7. Rev-Erb heterozygosity modulates metabolic gene expression in brown adipose tissue (BAT). (a) Mean expression of the glycolytic factors *Pfk1, Eno1, Hk1, Hk2, Tpi1* **(b)** Mean expression of the fatty acid oxidation factors *Cpt1a, Cpt1b, Ucp1, Ucp2* and *Ucp3.* mRNA was isolated from BAT using Trizol extraction from 15-week-old *Nr1d1*$^{+/+}$, *Nr1d1*$^{-/-}$ and *Nr1d1*$^{+/-}$ mice (n = 6). Gene expression was determined by RT-qPCR. *$p<0.05$ and **$p<0.01$ were determined by One-Way ANOVA. Data represented as a mean ± s.e.m.
